# Enteric pathogen carriage in early childhood is associated with elevated CRP, lower IGF-1 and linear growth deficits: the ELICIT study in rural Tanzania

**DOI:** 10.1136/bmjgh-2024-018454

**Published:** 2025-11-12

**Authors:** Mark D DeBoer, Sarah E Elwood, Godfrey Guga, Samwel Jatosh, Rebecca J Scharf, Jie Liu, Elizabeth T Rogawski McQuade, Estomih R Mduma, Eric Houpt, James A Platts-Mills

**Affiliations:** 1 Pediatrics, University of Virginia School of Medicine, Charlottesville, Virginia, USA; 2 Medicine, University of Virginia School of Medicine, Charlottesville, Virginia, USA; 3 Haydom Global Health Research Centre, Haydom Lutheran Hospital, Mbulu, Manyara, Tanzania, United Republic of; 4 Department of Epidemiology, Emory University, Atlanta, Georgia, USA; 5 Medicine, University of Virginia Health System, Charlottesville, Virginia, USA

**Keywords:** Child health, Paediatrics, Public Health

## Abstract

**Introduction:**

Globally, enteric pathogens have been associated with poor child growth and development. Our aim was to assess for predictors, consequences and potential mechanisms of enteric pathogen burden morbidity among children in rural Tanzania.

**Methods:**

We performed a secondary data analysis of 1140 children from the Early-Life Interventions for Childhood Growth and Development in Tanzania (ELICIT) study, assessing stool samples from ages 6, 12 and 18 months using TaqMan Array Cards for enteric pathogens. We examined risk factors for infections and associations of pathogen burden with serum biomarkers of nutrition and inflammation and anthropometry and cognitive development outcomes (using the Malawi Developmental Assessment Tool, MDAT).

**Results:**

Over the three time points, children had median (IQR) pathogen counts of 2 (0–7) at 6 months, 3 (0–8) at 12 months and 2 (0–8) at 18 months—for an overall mean of 7.79 total pathogen detections (SD 2.64, range 1–18). Assessed as an outcome, pathogen burden was associated with lower socioeconomic status, higher household occupancy and housing chickens. Assessed as a predictor (and adjusted for sex, socioeconomic status, birth month and age), each additional pathogen was associated with 0.03 lower length-for-age z-scores (p=0.008) and 0.04 lower weight-for-age z-scores (WAZ) (p<0.0001) by 18 months—with multiple individual pathogens analysed separately also being associated with lower WAZ. Pathogen burden was not associated with 18-month MDAT scores. In evaluating for possible mechanisms of growth suppression, pathogen burden was positively associated with serum C reactive protein and FGF21 and inversely associated with Collagen X and IGF-1 at 12 and 18 months—associations that were also all noted with *Shigella* infections individually.

**Conclusions:**

Despite the ubiquity of pathogen carriage, we noted important predictors and outcomes of greater pathogen burden—underscoring ongoing importance of identifying interventions to reduce pathogen exposure among children in developing areas.

**Trail registration number:**

NCT03268902.

**WHAT IS ALREADY KNOWN ON THIS TOPIC**

- Young children in low-resource areas are exposed to enteric pathogens that are related to linear growth deficits.
- The Early-Life Interventions for Childhood Growth and Development in Tanzania study assessed intervention with antimicrobials, demonstrating that while individual pathogens were reduced transiently, children were recolonised by 3 months later and the intervention did not improve childhood growth by 18 months.

**WHAT THIS STUDY ADDS**

- This study provides a comprehensive view of both predictors of total enteric pathogen carriage and downstream sequelae of growth and biomarkers.
- Of particular note is that the total pathogen burden was associated with elevations in inflammation as measured by systemic C reactive protein and low IGF-1 and collagen X, as well as clear deficits in linear growth, weight and head circumference.
- Carriage of *Shigella* alone was associated with all of these findings of total pathogen count, except for linear growth deficits.

**HOW THIS STUDY MIGHT AFFECT RESEARCH, PRACTICE OR POLICY**

- The predictors of higher pathogen carriage (lower maternal education, denser housing occupancy, unimproved water source) identify particular programmes governments could target.
- Research efforts should continue to focus on interventions to reduce pathogen exposure, lessen inflammation and improve nutrition.

## Introduction

Globally, over 148 million children under age 5 years continue to experience stunting of growth due to nutritional and infectious causes.^1^ Stunting is, in turn, associated with a decrease in human capital, with poorer linear growth being associated with lower cognitive status.^2^ One contributor to poor linear growth in young children has been early and repeated enteric infections, which are common both in the setting of symptomatic infections with diarrhoea^3^ and acute malnutrition^4^ and also in asymptomatic cases,^5 6^ with multiple individual pathogens being associated with lower length-for-age z-scores (LAZ) in each of these settings.^5 6^

Enteric pathogen carriage is thought to occur through repeated exposure from the surrounding environment,^7^ though attempts to address this via interventions to improve water supply and sanitation have not been successful at improving growth or reducing carriage.^8^,^10^ Exposure to enteric pathogens contributes to environmental enteric dysfunction, in which chronic exposure leads to impaired intestinal epithelial barrier with reduced nutrient absorption and potential translocation of pathogens.^11^ The presence of enteric pathogens is associated with markers of both enteric inflammation and systemic inflammation such as C reactive protein (CRP).^6^ This is then thought to lead to suppression of growth factors, resulting in poor growth.^6^ However, while lower IGF-1 levels have been noted among children with poor growth in developing areas,^4 12 13^ it has been unclear whether this is directly linked to a higher pathogen burden in young children as a potential mechanism for growth suppression.

We previously reported on an intervention delivering antimicrobials to a cohort of children in an area with high enteric pathogen carriage.^14^ Children who received azithromycin as a single dose on months 6, 9, 12 and 15 of life and a 3-day course of nitazoxanide (vs placebo) had a temporary reduction in pathogenic organisms *Campylobacter jejuni/coli*, *Shigella*/EIEC, enteroaggregative *Escherichia coli* (EAEC), and typical enteropathogenic *E. coli* (tEPEC) by 2 weeks after dosing but had a similar level of pathogen carriage 3 months after treatment.^15^ Moreover, we reported that this intervention did not result in improvement in linear growth, with 50% of children remaining stunted by age 18 months.^14^

Given the ongoing needs to characterise the effects of pathogen burden and identify possible means for prevention, our goal in the current analysis was to evaluate for potential contributors to enteric pathogen carriage and potential downstream sequelae. We hypothesised that close exposure to animals, denser housing and improved sanitation would be significant predictors and that higher pathogen carriage would be associated with systemic inflammation, suppressed growth factors and worsened growth and cognitive development. These data may have implications for addressing growth deficits in developing areas.

## Methods

Early-Life Interventions for Childhood Growth and Development in Tanzania (ELICIT) was a 2×2 factorial randomised controlled trial designed to assess the effects of two separate interventions (daily nicotinamide and scheduled antimicrobials) on childhood growth from age 0 to 18 months.^14^ The study design, participant baseline characteristics and primary outcomes have been reported previously.^14^,^17^ Inclusion criteria at enrolment were maternal age ≥18 years, child age <14 days, and the family’s stated intent to reside within a 25 km radius of Haydom Lutheran Hospital for the duration of the study. Exclusion criteria were multiple gestation, significant birth defect or neonatal illness, infant weight <1500 g at enrolment and lack of intent to breastfeed.

### Patient and public involvement

Patients or the public were not involved in the design, or conduct, or reporting, or dissemination plans of our research.

### Interventions

As part of the factorial design, approximately equal groups of participants received either nicotinamide and antimicrobials, placebo for both arms, nicotinamide and placebo, or antimicrobial and placebo.^14 16^ For the nicotinamide intervention, lactating mothers received a nicotinamide 250 mg tablet daily (or placebo) during months 0–6 (hypothesised to be conferred to the child in the breast milk), and children received sachets of nicotinamide 100 mg (or placebo) mixed in their food daily from months 7–18. Nicotinamide and corresponding placebo were manufactured by Vita-gen (New York, USA).

For the antimicrobial intervention, participants received azithromycin or its corresponding placebo (both manufactured by Universal Corp, Kenya) as a single oral dose of 20 mg/kg of 200 mg/5 mL suspension at months 6, 9, 12 and 15.^14 16^ Nitazoxanide or placebo (both by Romark, Florida, USA) was administered as a 3-day course of 100 mg two times per day of 100 mg/5 mL suspension at months 12 and 15.

### Data collection

All study visits occurred at the family’s home, including enrolment and monthly visits. Child length, weight and head circumference were measured at enrolment and every 3 months thereafter. At these visits, length was assessed using measuring boards, weight was assessed using digital scales and head circumference was assessed using measuring tape, with additional details described previously.^14 16^ All anthropometry measures were converted to WHO z-scores, including LAZ, weight-for-age (WAZ) and head-circumference-for-age (HCZ).

At each monthly visit, mothers responded to questionnaires including healthcare utilisation, medications and child illness symptoms, such as whether in the past week^18^ the child had illness, a fever, a cough or diarrhoea. Mothers were additionally asked whether in the past week the child had eaten any dairy, meat, eggs and/or beans (legumes). At the first monthly visit, mothers responded to questions about demographic and economic factors. Participant socioeconomic status (SES) was assessed using a scoring system developed from a prior study (MAL-ED) based on improved water and sanitation, assets, maternal education and household income (WAMI).^19^

### Stool testing

Stool samples were collected at 6, 12 and 18 months and stored at −80°C until time of testing. Procedures for sample extraction and testing have been previously detailed^20 21^ and the protocols are available at dx.doi.org/10.17504/protocols.io.5qpvo3k8xv4o/v1. We used custom-designed TaqMan Array Cards (TAC)(ThermoFisher, Carlsbad, California, USA) that compartmentalised probe-based quantitative PCR assays for multiple enteropathogens (online supplemental table 1). A cycle threshold of 35 was used as the limit of detection and any detections below that were considered positive. Pathogens with >5% prevalence in the cohort were included in the analysis. Bacteriophage MS2 and phocine herpesvirus were used as external controls to monitor efficiency of nucleic acid extraction and amplification. We included one blank per extraction batch and one no-template amplification control per ten cards to exclude laboratory contamination.^15^

### Measurement of circulating biomarkers

At the 12-month and 18-month visits, haemoglobin levels were assessed using the Hemocue Hb 201+point-of-care device according to the manufacturer’s specifications (HemoCue America, Lyndhurst, New Jersey, USA). The child’s finger was cleansed and pricked with sterile lancet, and blood was placed in a cuvette in the HemoCue device.

Blood samples were taken via phlebotomy at 12 and 18 months and separated into serum and stored at −80°C until time of testing. Collagen-X levels were assessed at Shriner’s Hospital, Portland, OR via ELISA as described previously.^22^ The Micronutrient and Environmental Enteric Dysfunction Assessment Tool was performed by PATH (Seattle, Washington, USA) as previously described.^23^ This multiplex bead system assessed for levels of IGF-1, FGF21, thyroglobulin, ferritin, sTFR, RBP4, CRP, AGP and CD14 (online supplemental table 2). Serum samples were assessed on a Q-Plex array (Quansys Biosciences, Utah, USA) which concurrently quantifies multiple markers using geometric planar arrays of marker-specific antibodies in each well of a 96-well plate. Samples were combined with diluent and detection mix in a 96-well plate and imaged using a Q-View Imager LS (Quansys Biosciences). Samples below the lower limit of quantification (LLOQ) were assigned a value just below the LLOQ.

### Cognitive development assessment

At the 18-month visits, trained personnel administered the Malawi Developmental Assessment Tool (MDAT), a cognitive scale created to assess child development in children living in a rural sub-Saharan context. The MDAT is a relatively new assessment (published in 2010) with good reliability in sub-Saharan Africa (94%–100%).^24^ Further details on the use of this scale in ELICIT have been published previously.^25 26^ Briefly, MDAT is a neurodevelopmental scale for use in early childhood, with the following subscores: fine motor, gross motor, language and social. The ELICIT cognitive team worked together to adapt, pilot and validate the MDAT locally for Haydom. The items, words and tasks used in the assessment were similar to those developed in the original study environment in rural Malawi. Six field workers completed 4 weeks of training and practice. The assessment was delivered in Swahili or Iraqw, the first language for most participants. The fine and gross motor items did not require any adaptation and were used as developed in Malawi. Several questions in the language and social domains required adaptations for words not directly translatable to Swahili. Items were scored as 1 (pass) or 0 (not pass). Within each subtest, children completed items until they received a score of 0 for six items in a row. Standardised MDAT Z-scores were calculated using version 1.1 of the MDAT Scoring Application.

### Statistical analysis

Statistical analyses were performed using SAS V.9.4 or R V.4.2.1 (with mediation package 4.5). Analyses were performed using all available data (including all available blood samples) from the RCT. For factors that were non-normally distributed, analyses were performed using log-transformed values. TAC data for each pathogen represented whether that pathogen was present or absent for that participant at a given time point. The number of pathogens present was totalled at each time point for each participant. For participants who had data on pathogen number available at all three time points, these were combined for that participant to yield a total study pathogen count. Biomarkers were assessed for normalcy and log-based values were used for those not normally distributed. Biomarker values were then standardised with a mean of 0 and an SD of 1.0 such that all regression coefficients reflect relationships for each 1 SD. We performed mixed-model linear and logistic regression assessing associations between the number of enteric pathogens (at each time point—months 6, 12, 18—as the independent variable) modelled as ‘random intercept’ with outcomes including biomarkers, symptoms of illness, cognitive status and anthropometry measure. Adjusted models included sex, SES (WAMI),^19^ birth month, age and baseline value (when appropriate) as additional variables. The central analysis assessed the relationship between pathogen burden and LAZ using adjusted mixed-model linear regression. We also assessed the associations of each of the individual pathogens with these outcomes using similar mixed-model regressions for each of the individual pathogens. We performed linear and logistic regression at individual time points (months 6, 12 and 18) to assess for temporal trends. Prior assessments of randomisation subgroups for the interventions of this RCT failed to show differences in outcomes, including anthropometry, MDAT scores and circulating biomarkers, and were not included in the models. As a sensitivity analysis, we assessed our central analysis for each of the four 2×2 factorial treatment groups.

We used formal mediation analysis for any biomarkers associated with pathogen sum, first assessing linear mixed effects models to evaluate the relationship between exposure (pathogen sum) and the hypothesised mediator (each of the biomarkers) adjusted for sex, WAMI, lagged anthropometry and birth season with a random slope per participant. We then assessed similar models to look at the effects of pathogen sum on the anthropometry of interest (LAZ, WAZ and HCZ) adjusted by the respective biomarker and all other variables in the prior model. We then used the mediation package in R to compare those two models and determine the average causal mediation effect and the proportion mediated.

As performed previously,^13^ because of the large number of biomarkers and individual pathogens tested, for analyses involving the entire set of these biomarker and pathogen variables, we used a false discovery rate approach to adjust the p value used to determine statistical significance for these tests. For an analysis with 11 biomarkers, (eg, p values were ranked in order, smallest to largest, p(1) < p(2) < p(3)…. < p(11)). For each p value, statistical significance was determined by computing 0.05*i/11. Thus, significance for p(1) was set at 0.05*1/11=0.004545; p(2) was set at 0.05*2/11=0.00909, etc (online supplemental table 3). We then found the first p value where p(i) exceeded its calculated significance threshold value. All p values prior to this were declared ‘significant by false discovery rate approach’.^27^ For other analyses, a p<0.05 was used to determine statistical significance.

## Results

### Participant characteristics

We assessed data from 1140 participants with available TAC data, with characteristics as shown in table 1. Participants reflected the low-resource nature of the area around Haydom; for example, only 1.4% had a flush toilet. At 6, 12 and 18 months, diarrhoeal symptoms over the past week were reported for 7.6%, 7.9% and 5.6% and non-study antimicrobials were used over the past month in 1.9%, 15.2% and 11.8%, respectively. Finally, as reported previously, there were significant growth deficits, with 49.2% of children being stunted by age 18 months but with very little wasting (prevalence of weight-for-length z-score <−2=2.6%).

**Table 1:** Participant characteristics*

	Participants
Sex—Males	51% (558/1140)
SES	
Maternal education (years)	6.37 (3.05)
Household income (monthly, in USD)	US$12.90 (US$8.60, US$21.50)
WAMI	0.305 (0.130)
Housing density	
Number of siblings at home	3 (2, 5)
Number of people in home	7 (5, 9)
Water/hygiene resources (%)	
Improved latrine	11.1% (126/1140)
Flush toilet in home	1.4% (16/1140)
Access to improved water source	66.2% (755/1140)
Water piped into dwelling	0.5% (6/1140)
Animals at home (%):	
Chickens	87% (836/965)
Cows	68% (521/770)
Pigs	63% (225/356)
Family owns agricultural land	97% (1105/1140)
Illness symptoms/medical care in past week (%) at 6/12/18 months	
Illness	8.3%/12.2%/10.7%
Diarrhoea	7.6%/7.9%/5.6%
Fever	7.7%/6.0%/4.5%
Sought medical care	20.9%/22.5%/18.6%
Antimicrobial use (past month)	1.9%/15.2%/11.8%
Growth at 6/12/18 months	
Stunted, (LAZ-Z <−2)	23.5%/37.0%/49.2%
WAZ <−2	7.5%/10.9%/13.2%
HCZ <−2	5.4%/ 5.1%/3.2%
Wasted, (WFL-Z <−2)	3.9%/2.3%/2.6%

* For continuous variables, mean (SD) and median (IQR) are provided. For proportions, percentage of participants with available data are shown.

HCZ, head circumference-for-age z-score; LAZ-Z, length-for-age z-scores; SES, socioeconomic status; WAMI, water, assets, maternal education, income; WAZ, weight-for-age z-score; WFL-Z, weight-for-length z-score.

### Pathogen burden

**Figure 1:**
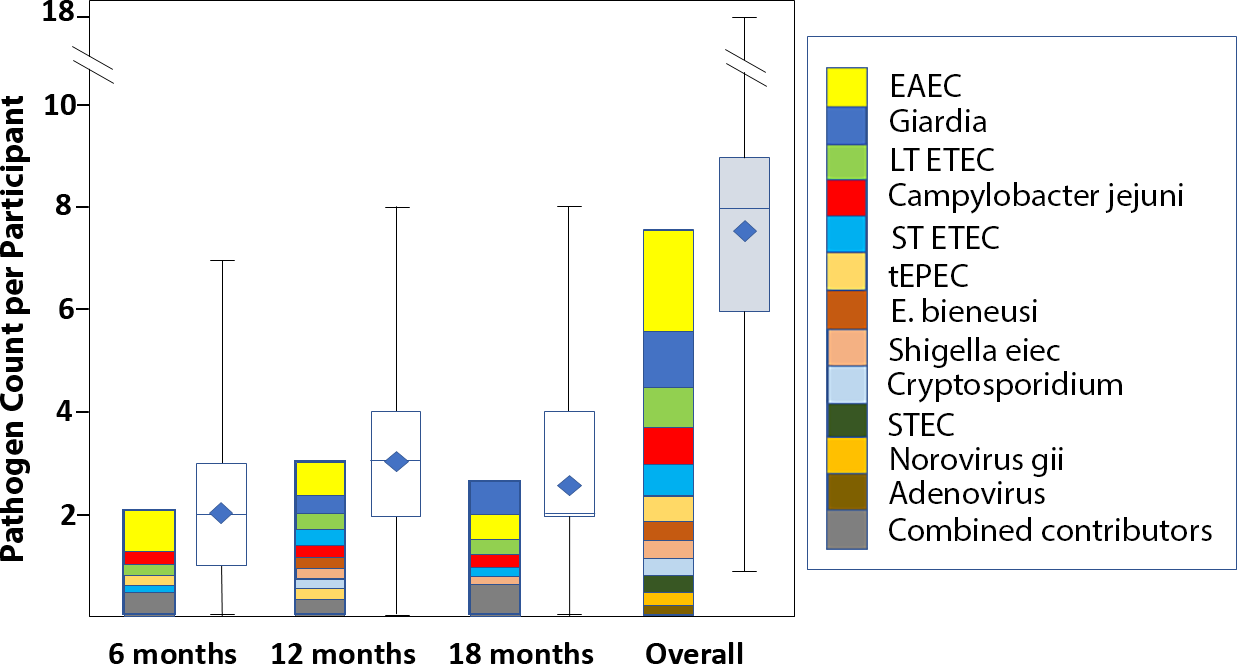
Total number of pathogens detected at 6, 12 and 18 months and overall. 12 pathogens had prev>5% and were included in the analysis. Shown side by side are stacks of mean prevalence by organism (with those with prevalence <14% at that time point shown in grey as ‘combined contributors’) and box plot with mean (blue diamond), median (centre line) and range. EAEC, enteroaggregative *Escherichia coli*; *E. bieneusi*, *Enterocytozoon bieneusi*; EIEC, enteroinvasive *E*. *coli*; ETEC, enterotoxigenic *E*. *coli*; STEC, Shiga toxin-producing *E. coli*; tEPEC, typical enteropathogenic *E. coli.*

12 pathogens had an overall prevalence >5% and were included in the analysis. Prevalence of individual pathogens by time point and combined pathogen burden is displayed in figure 1 and online supplemental table 4 and online supplemental figure 1. Of the 1140 participants included in the analysis, TAC data were missing for 80 at 6 months, 162 at 12 months and 111 at 18 months. SES as measured by WAMI was not different at any time point between those who were or were not missing TAC results (using t-tests, all p>0.250).

Participants had a mean number of pathogens of 2.07, 2.99 and 2.64 at months 6, 12 and 18, and a mean cumulative 7.65 pathogens per participant over the three time points. The most common pathogens were EAEC, *Giardia* and *Campylobacter jejuni/coli*, which were found a cumulative 1.94, 1.04 and 0.93 times per participant over the three time points.

### Potential contributors to pathogen burden

Factors evaluated as potential contributors to pathogen burden are displayed in table 2. Participants randomised to the antimicrobial arm (for which administration began after the 6-month stool sample was collected) had a slightly higher pathogen burden at the 12-month and 18-month time points (5.95 (2.25) vs 5.55 (2.05), p=0.009). Neither the study nicotinamide nor recent use of non-study antimicrobials was associated with overall pathogen burden. Sex was also not associated with number of pathogens. From an SES perspective, both the SES estimate WAMI and two of its individual components of maternal education and family income were inversely associated with pathogen burden, while the number of people living in the household was positively associated. Pathogen burden was also associated with access to an improved water source but not access to improved latrine. With respect to animals (who in this region often spend time inside the house), pathogen burden was higher among children whose families had chickens at home, an association not seen among families with cows, goats or pigs at home.

**Table 2:** Predictors of combined stool pathogen sum from months 6, 12 and 18*

Characteristic	Mean difference (SE)	P value
Medications/interventions		
Antimicrobial intervention†	0.387 (0.097, 0.676)	**0.009**
Nicotinamide intervention	−0.049 (−0.407, 0.308)	0.786
Non-study antibiotic use within month before TAC testing	−0.140 (−0.414, 0.135)	0.315
Sex—Male	−0.034 (−0.392, 0.324)	0.853
Seasonality		
Preharvest	0.339 (−0.026, 0.704)	0.068
Socioeconomic status		
Maternal education (years)	−0.067 (−0.125,–0.008)	**0.026**
Family income (USD)	−0.011 (−0.019,–0.003)	**0.005**
WAMI‡	−0.462 (−0.640,–0.284)	**<0.0001**
Housing density		
Number of siblings at home	0.042 (−0.036, 0.120)	0.294
Number of people in home	0.076 (0.012, 0.139)	**0.019**
Nutrition§		
Dairy	0.102 (−0.012, 0.216)	0.079
Meat	−0.044 (−0.157, 0.068)	0.437
Eggs	0.082 (−0.014, 0.178)	0.094
Beans	0.137 (−0.054, 0.328)	0.159
Water/sanitation		
Improved latrine	−0.068 (−0.214, 0.077)	0.357
Flush toilet	−1.590 (−3.325, 0.146)	0.073
Access to improved water source	−0.177 (−0.271,–0.083)	**<0.001**
Water piped into dwelling	−1.301 (−3.900, 1.298)	0.326
Animals kept at home:		
Chickens	0.730 (0.166, 1.295)	**0.011**
Cows	0.245 (−0.218, 0.709)	0.299
Pigs	0.202 (−0.469, 0.873)	0.554
Family owns agricultural land	0.647 (−0.427, 1.720)	0.238

Bolded p values are considered statistically significant.

* Assessed as sum of months when child was reported to have eaten a given food type in the week prior to monthly assessment for months 6–18, with regression adjusted for 6 month pathogen count.

† Adjusted for 6 month value, which was prior to antimicrobial administration.

‡ This score has been standardised for the cohort, to have mean of 0 and SD of 1.

§ Assessed using linear regression except non-study antibiotic use (mixed model assessing antibiotic use and sum of pathogens at months 6, 12 and 18).

TAC, TaqMan Array Cards; WAMI, water, assets, maternal education, income.

We further assessed potential contributors to individual pathogens (online supplemental table 5a, b). Reported use of non-study antimicrobials in the prior month had a positive association with EAEC detection but negative association with *Giardia* (both measured using mixed model analysis assessing non-study antimicrobials and pathogen carriage at any given month). Multiple pathogens were inversely related to SES characteristics.

### Potential sequelae to pathogen burden

Monthly pathogen burden at 6, 12 and 18 months (assessed as mixed model) was associated with reported diarrhoea in the past week but not recent fever, illness, seeking healthcare or being hospitalised (table 3; individual time point relationships in online supplemental table 6). This appeared to be driven by the 18-month time point, as pathogen sum was similar between those with and without diarrhoea at months 6 and 12 (online supplemental table 7a). Conversely, while those with pathogen counts in the upper tertile were more likely than those with lower pathogen counts to report diarrhoea at 12 and 18 months, those with a report of recent diarrhoea still represented at most 10.3% of those with higher pathogens—underscoring that most pathogen carriage was asymptomatic (online supplemental table 7b).

**Table 3:** Associations of enteric pathogen burden with recent illness symptoms, anthropometry and cognitive development

	Unadjusted*		Adjusted for sex, WAMI, birth month, age*	
		P value		P value
Reported symptoms in prior week at 6, 12, 18 months†	OR (95% CI)		OR (95% CI)	
Illness	1.02 (0.95 to 1.08)	0.635	1.03 (0.97 to 1.11)	0.348
Diarrhoea	1.13 (1.03 to 1.24)	**0.013**	1.13 (1.02 to 1.25)	**0.021**
Fever	0.99 (0.90 to 1.11)	0.915	1.07 (0.95 to 1.19)	0.265
Sought medical care	1.04 (0.98 to 1.11)	0.207	1.05 (0.99 to 1.13)	0.115
Hospitalised	1.20 (0.93 to 1.55)	0.167	1.03 (0.97 to 1.11)	0.348
Anthropometry at 6, 12 and 18 months‡	Mean difference (SE)		Mean difference (SE)	
LAZ	−0.083 (−0.104 to –0.061)	**<0.0001**	−0.027 (−0.047 to –0.007)	**0.008**
WAZ	−0.070 (−0.081 to –0.048)	**<0.0001**	−0.039 (−0.055 to –0.023)	**<0.0001**
HCZ	−0.041 (−0.055 to –0.022)	**<0.0001**	−0.038 (−0.056 to –0.020)	**<0.0001**
Cumulative pathogen total on 18-month anthropometry	Mean difference (SE)		Mean difference (SE)	
LAZ	−0.035 (−1.683 to –1.296)	**0.003**	−0.029 (−0.052 to −0.007)	**0.011**
WAZ	−0.042 (−0.065 to –0.019)	**<0.001**	−0.037 (−0.060 to –0.013)	**0.003**
HCZ	−0.040 (−0.061 to –0.019)	**0.011**	−0.039 (−0.059 to –0.019)	**<0.001**
Extreme anthropometry measures at 6, 12 and 18 months§	OR (95% CI)		OR (95% CI)	
Stunted (LAZ<−2)	1.13 (1.07 to 1.20)	**<0.0001**	1.05 (0.98 to 1.13)	0.141
Low weight (WAZ<−2)	1.13 (1.03 to 1.23)	**0.008**	¶	
Wasted (WFL-Z <−2)	1.00 (0.86 to 1.16)	0.987	¶	
Cumulative pathogen total on 18-month cognitive development	Mean difference (SE)		Mean difference (SE)	
MDAT	0.006 (−0.013 to 0.026)	0.564	0.001 (−0.017 to 0.086)	0.938

Bolded p values are considered statistically significant.

* Anthropometry measures in all cases are further adjusted for baseline measure. MDAT assessment further adjusted for age of assessment.

† Odds of illness symptoms assessed using mixed model (glimmix logit).

‡ Anthropometry z-scores assessed using linear regression mixed model further adjusted for age at measurement.

§ Odds of extreme measure using mixed model, further adjusted for age at measure.

¶ Mixed model regression did not converge.

HCZ, head circumference-for-age z-score; LAZ, length-for-age z-score; MDAT, Malawi Developmental Assessment Tool; WAMI, water and sanitation, assets, maternal education and household income; WAZ, weight-for-age z-score; WFL-Z, weight-for-length z-score.

Pathogen burden was inversely associated with LAZ, WAZ and HCZ—relationships that remained after adjustment for sex, SES, age and birth month (table 3). When assessed as the association between cumulative pathogen level at 18 months and anthropometry measures at 18 months in adjusted models, each added pathogen was associated with a decrease in z-score of 0.029, 0.037 and 0.038 for LAZ, WAZ and HCZ, respectively. Over the range of cumulative pathogen burden by age 18 months (1–18 pathogens), this represented differences of approximately 1.4 cm in length and 0.8 kg in weight. With respect to study power, we had 80% power to detect an effect size of 0.166 difference in anthropometry z-score between groups that had fewer (vs greater) than the median number of pathogens.

Pathogen burden was associated with increased odds of stunting, but this was attenuated in the adjusted model (table 3). In a sensitivity analysis to assess any potential effect of the original 2×2 factorial RCT design, these relationships between pathogen burden and LAZ persisted in all four randomisation subgroups, all with parameter estimates −0.020 and −0.049 per added pathogen.

Cognitive development, as assessed using MDAT, was not associated with pathogen burden. Given the overall sample size of 1140 and MDAT SD of 0.15, we had 80% power to detect as little as a 0.0225 difference in MDAT score.

We also assessed for associations with outcomes for individual pathogens, shown in online supplemental table 1a, b). *Shigella/*EIEC and *Cryptosporidium* were associated with reported recent diarrhoea while EAEC was associated with having recently sought medical care. EAEC, *Giardia* and *Shigella* were associated with both lower WAZ, while *Shigella* was additionally associated with lower HCZ. While use of non-study antimicrobials overall was not associated with LAZ (online supplemental table 8c), when assessing contributions from individual types of antimicrobials, use of non-study penicillins was associated with greater LAZ at 18 months (0.066 (0.019) p<0.001).

## Associations with circulating biomarkers

Finally, to provide potential insights into mechanisms behind growth-related associations, we assessed for associations with biomarkers related to nutrition and inflammation, using standardised values to assess relative associations (table 4). From a nutrition standpoint, pathogen burden was inversely associated with IGF-1 (point estimate −0.08, p<0.0001) and Collagen X (−0.04, p=0.019) and positively associated with FGF21, a marker of hepatic nutrient deprivation (0.06, p<0.001)(table 3). From an inflammatory perspective, pathogen burden was positively associated with systemic CRP (0.05, p=0.002) and inversely associated with RBP4, which is involved with vitamin A transport but is also a marker of inflammation (−0.4, p=0.017). We also assessed for these associations at individual time points, as shown in online supplemental table 6, revealing that associations at 18 months more closely paralleled those seen in the mixed model. Given the overall sample size of 1140 and an SD of 1.0 for each of the standardised biomarkers, we had 80% power to detect as little as a 0.083 difference for those above and below the median pathogen burden.

**Table 4:** Associations of enteric pathogen burden with biomarkers at 12, 18 months

	Unadjusted		Adjusted for sex, WAMI, birth month, age at measure	
Biomarker*	Standardised mean difference (SE)	P value†	Estimate (SE)	P value†
Haemoglobin	0.001 (−0.030 to 0.032)	0.938	−0.002 (−0.034 to 0.029)	0.885
Collagen X	−0.033 (−0.069 to 0.003)	0.069	−0.040 (−0.073 to –0.007)	**0.019**
IGF-1	−0.079 (−0.108 to –0.049)	**<0.0001**	−0.080 (−0.108 to –0.051)	**<0.0001**
FGF21	0.054 (0.021 to 0.086)	**0.001**	0.060 (0.028 to 0.092)	**<0.001**
Thyroglobulin	−0.012 (−0.043 to 0.018)	0.425	−0.006 (−0.036 to 0.023)	0.669
Ferritin	0.006 (−0.026 to 0.038)	0.726	−0.002 (−0.033 to 0.029)	0.909
sTFR	−0.007 (−0.037 to 0.022)	0.620	−0.010 (−0.039 to 0.019)	0.502
RBP4	−0.040 (−0.072 to –0.008)	**0.015**	−0.037 (−0.068 to –0.007)	**0.017**
CRP	0.049 (0.017 to 0.081)	**0.003**	0.051 (0.020 to 0.083)	**0.002**
AGP	0.033 (0.001 to 0.065)	0.045	0.027 (−0.004 to 0.058)	0.091
CD14	0.014 (−0.016 to 0.044)	0.363	0.007 (−0.024 to 0.038)	0.650

Bolded p values are considered statistically significant.

* Biomarkers that were not normally distributed were converted to log-based scale. All biomarkers were then standardised with a mean of 0 and SD of 1.

† Statistically significant by False Discovery Rate (see online supplemental table 3, 11 outcomes).

CRP, C reactive protein; WAMI, water, assets, maternal education, income.

We next used a formal causal mediation analysis to assess the biomarkers associated both TAC pathogen burden for their contribution to anthropometry outcomes also linked to pathogen burden (online supplemental table 8). In this analysis, none of the biomarkers was a significant mediator of LAZ or HCZ, while IGF-1, FGF21 and collagen X were all significant mediators of WAZ, with proportions attributed of 0.15, 0.20 and 0.08, respectively (online supplemental table 8).

In evaluating for associations with individual pathogens (online supplemental table 9a, b), *Shigella* was the organism with the greatest number of associations with biomarkers, being associated with much stronger effect sizes as seen in the total pathogen model, including suppressions in IGF-1 (−0.22, p<0.0001) and collagen X (−0.20, p=0.001) and higher levels of FGF21 (0.23, p<0.001), CRP (0.16, p=0.011) and AGP (0.14, p=0.019). *Giardia* was the only other organism associated with elevated FGF21 (0.175, p<0.001) and was also associated with elevations in CD14 (0.156, p=0.002). *Cryptosporidium* and Norovirus were associated with suppressed IGF-1 levels (−0.15, p=0.009 and −0.32, p<0.0001, respectively), while *E. bieneusi* was inversely associated with RBP4 (−0.26, p<0.0001) (online supplemental table 8a, b).

### Discussion

We noted a high prevalence of enteric pathogen carriage in this cohort of young children in rural Tanzania, with children having a cumulative 1–18 pathogen detections over three time points—and with a higher pathogen burden associated with growth deficits. In most cases, this occurred without observed diarrhoea, underscoring the challenge of detecting and treating enteric infections in these populations. These growth deficits coincided with positive associations between pathogen burden and systemic inflammation as measured by CRP and suppression of the growth factor IGF-1, highlighting a potential mechanism, as we had previously noted inverse associations between CRP and IGF-1, and positive associations between IGF-1 and LAZ (online supplemental figure 2).^13^ Finally, pathogen burden was associated with denser housing occupation and chickens at home—identifying potential areas to address in efforts at prevention in affected areas.

The main underlying hypothesis of this RCT was that in an area where children have a high prevalence of enteric pathogen carriage, intervention with antimicrobials covering multiple pathogens would reduce carriage and thus improve growth. In this intervention, participants received a dose of azithromycin (or placebo) every 3 months starting at age 6 months (targeting *Campylobacter*, *Shigella* and multiple pathogenic *E. coli* species) and a 3-day course of nitazoxanide at 12 and 15 months (covering *Giardia* and *Cryptosporidium*). However, we previously reported that not only did we not see improved growth among those randomised to receive antimicrobials, we did not see sustained reduction in pathogen burden.^15^ By 2 weeks after antimicrobial dosing, there were short-term reductions in *Campylobacter jejuni/coli*, EAEC, *Shigella/*EIEC and tEPEC; however, by 3 months later, there was a return of pathogens.^15^

Interestingly, this is the first analysis for this study in which we report that participants who received non-study penicillins had higher LAZ, with adjusted models showing a positive correlation between the cumulative number of penicillin courses and 18-month LAZ. This contrast in association with higher LAZ between the intervention antimicrobials and non-study may relate to (1) the lower antimicrobial exposure for the intervention (one dose of azithromycin every 3 months) compared with non-study use (presumably a complete course of treatment) or (2) the difference in antimicrobial used, with penicillins potentially having been more effective in this setting. Still, use of all non-study antimicrobials was only associated with a decrease in *Giardia*—and was associated with an increase in prevalence of EAEC, potentially due to reverse-causation, as we cannot determine whether these children were placed on antimicrobials because of their symptoms from EAEC.

Also interesting was that among children randomised to the antimicrobial arm, there was a slightly higher total pathogen count for months 12 and 18 (after the antimicrobial intervention began), with an increase of 0.4 pathogens between the two time points. The cause of this is uncertain, though this may suggest potential effects on reducing commensal bacteria following administration, permitting colonisation by a greater variety of pathogens. The same association was not seen with non-study antimicrobials, which were presumably administered because of symptoms potentially produced from pathogens, as supported by online supplemental table 8c).

The recolonisation of pathogens after the antimicrobial intervention demonstrated from our prior study suggests continued environmental exposure to these pathogens. In addition to animal-associated exposures—with chickens being a previously reported risk factor^11^—water contamination and a lack of hygiene are a common source of re-exposure in many developing areas, as we noted in lower pathogen burden among families using improved water sources. The association of pathogen count with number of people in the house could be a further link to cumulative exposure to poor hygiene.^28^ Nevertheless, it should be noted that large-scale studies to improve water sources and instal latrines were not successful in improving growth or reducing pathogen carriage,^8^,^10^ emphasising the complexities of attempting to reduce ongoing pathogen exposure.

IGF-1 is the main growth factor in the body—released primarily from the liver in response to growth hormone, with expression affected by both nutrition and inflammation.^29^,^31^ We had previously reported from cohorts in low-resource settings in Brazil that highlighted how symptoms of illness and systemic inflammation are associated with growth hormone resistance, in which serum levels of CRP are correlated with higher growth hormone but lower IGF-1.^32^ Maleta *et al* noted lower IGF-1 levels in Malawian children with *Shigella*, *Campylobacter*, enterovirus and malaria infections.^33^ The current study expands on these concepts in seeing IGF-1 suppression also in children with *Cryptosporidium* infections—and in the case of *Shigella,* additional associations with deficits in WAZ. We further found that *Giardia* and *Shigella* were associated with both higher levels of FGF21—a hepatic marker of inadequate nutrition—and higher inflammation as assessed by CRP and AGP in the case of *Shigella* and as assessed by elevated CD14 in the case of *Giardia*. Importantly, the majority of these children did not have reported symptoms of illness, but by virtue of colonisation with pathogens still had higher systemic inflammation and lower IGF-1, as potential explanation for their linear growth deficits, though formal mediation analysis revealed mediation associations between IGF-1 and WAZ, not LAZ. We were unable to evaluate mechanisms for the increased systemic inflammation, but prior studies have suggested that pathogens cause a denuding of intestinal brush barrier, permitting translocation of bacteria and systemic inflammation.^11^

While we did not notice associations between pathogen burden and cognitive development, a prior study that included data from children around Haydom found that early childhood pathogen burden was associated with developmental deficits at age 8 years.^34^ That study both included a richer assessment of pathogen prevalence (assessing stool samples monthly for the first 2 years) and cognitive assessment at an older age (which provides a better assessment of future cognitive potential than early measures). Follow-up of the current cohort assessing cognition at an older age may help clarify potential long-term sequelae.

This study included detailed assessments of stool pathogens and related factors on a large cohort of children. Limitations of an analysis like this include the potential for confounding, particularly since there were inverse associations between maternal education and family income—which themselves could be associated with improved nutrition, for example. Still, we did not note attenuation of these associations after including an estimate of SES in the model. We lacked measures of nutritional intake that may have also contributed to growth outcomes—potentially helping to overcome some of the effects of pathogens. We only had pathogen measures at three time points and lacked assessment of circulating biomarkers at the 6-month time point, while more frequent assessments may have revealed more about timing windows of importance for these associations. We were missing TAC data for up to 14% of participants at each of the time points, though we did not find differences in SES between those who were or were not missing TAC results. Our primary analysis evaluated for relationships between the total pathogen count and outcomes—which treated all pathogens as potentially exerting equivalent associations, while assessments of the individual pathogens confirmed that some pathogens appeared more virulent than others. Finally, our observations regarding non-study antimicrobials—which were not randomised or systematically distributed—could also be heavily influenced by confounding factors, such as these families being more attentive to their child’s health.

In conclusion, we provided additional details on how enteric pathogens may adversely affect a child’s health. Individual pathogens did not exhibit consistent associations with outcomes, potentially suggesting these consequences are from a broader exposure to multiple pathogens over time. The slightly higher pathogen count among those randomised to receive scheduled antimicrobials warrants further assessment, including regarding effects on the overall microbiome. These data serve as an important reminder of the role of enteric pathogen carriage in developing areas of the world with risks for ongoing systemic inflammation and growth suppression—and the need for interventions to reduce exposures.

## Supplementary material

10.1136/bmjgh-2024-018454 online supplemental file 1

## Data Availability

Data are available on reasonable request.
