# Supplementary material for: Enteric pathogen carriage in early childhood is associated with elevated CRP, lower IGF-1 and linear growth deficits: the ELICIT study in rural Tanzania
Source: BMJ Glob Health. 2025 Nov 12;10(11):e018454. doi: 10.1136/bmjgh-2024-018454 (PMC12612773; doi:10.1136/bmjgh-2024-018454)

**ELICIT TAC Correlations: Supplemental Material**

Supplemental Table 1. Pathogens and gene targets tested by PCR reported in this study.

|  | Pathogen/target | Gene |
| --- | --- | --- |
| Viruses | Adenovirus 40/41 | Fiber gene |
|  | Norovirus GII | ORF1-2 |
| Bacteria | EAEC* | *aaiC, aatA* |
|  | Typical EPEC* | *bfpA* |
|  | LT ETEC* | *LT* |
|  | ST ETEC* | *STh, STp* |
|  | STEC* | *stx1, stx2* |
|  | *Campylobacter jejuni* and *C. coli* | *cadF* |
|  | *Shigella/*EIEC | *ipaH* |
| Fungi | *Enterocytozoon bieneusi* | *ITS* |
| Protozoa | *Cryptosporidium* | 18S rRNA |
|  | *Giardia lamblia* | 18S rRNA |

* *E. coli* pathotypes were defined as follows: EAEC (*aaiC*, or *aatA*, or both), typical EPEC (*bfpA*), LT ETEC (LT without STh or STp), ST ETEC (STh, or STp, or both), STEC (*eae* without *bfpA* and with *stx1*, *stx2*, or both).

Abbreviations: EAEC = enteroaggerative *E. coli*; EPEC = enteropathogenic *E. coli*; ETEC = enterotoxigenic *E. coli*; STEC = Shiga toxin-producing *E. coli*; EIEC = enteroinvasive *E. coli*.

Supplementary Table 2: Scientific rationale for assessing links between enteric pathogens and circulating biomarkers*

| Circulating biomarker | Testing modality | Health category | Rationale for assessing |
| --- | --- | --- | --- |
| Hemoglobin | Hemocue point-of-care testing | Nutrition | Required for oxygen transportation in blood; low levels associated with iron deficiency; can also decline during infections |
| Collagen-X | ELISA testing at Shriner’s | Nutrition | Associated with active growth of bone tissue |
| Insulin-like Growth Factor-1 (IGF1) | MEEDAT | Nutrition | Predominant growth factor; highly linked to nutrition; low levels frequently associated with growth hormone (GH) resistance |
| Fibroblast growth factor-21 (FGF21) | MEEDAT | Nutrition | Produced in liver during fasting; associated with GH resistance from low protein intake and adaptive starvation |
| Thyroglobulin | MEEDAT | Nutrition | High levels associated with iodine deficiency |
| Ferritin | MEEDAT | Nutrition &  Inflammation | Acute phase reactant during inflammation; low levels can indicate iron storage depletion |
| Soluble transferrin receptor (sTFR) | MEEDAT | Nutrition & Inflammation | Inversely associated with iron storage status; can also be associated with inflammation |
| Retinol binding protein-4 (RBP4) | MEEDAT | Nutrition & Inflammation | Involved in Vitamin A transport; high levels associated with inflammation |
| C-reactive protein (CRP) | MEEDAT | Inflammation | Acute phase reactant during inflammation |
| A1 glycoprotein (AGP) | MEEDAT | Inflammation | Acute phase reactant during inflammation |
| CD14 | MEEDAT | Inflammation | Marker of monocyte activation (e.g. LPS exposure) and Gram-negative bacterial translocation; associated with future growth faltering |

Abbreviations: MEEDAT: Micronutrient and Environmental Enteric Dysfunction Assessment Tool multiplex testing by PATH (Seattle WA, USA)

Supplementary Table 3: P-value cut-offs for false discovery rate approach.*

| For analyses involving 11 different outcomes | | For analyses involving 17 different outcomes | | For analyses involving 19 different outcomes | |
| --- | --- | --- | --- | --- | --- |
| Sequential P value order | False discovery rate P value cut off | Sequential P value order | False discovery rate P value cut off | Sequential P value order | False discovery rate P value cut off |
| 1 | 0.005 | 1 | 0.002941176 | 1 | 0.003 |
| 2 | 0.009 | 2 | 0.005882353 | 2 | 0.005 |
| 3 | 0.014 | 3 | 0.008823529 | 3 | 0.008 |
| 4 | 0.018 | 4 | 0.011764706 | 4 | 0.011 |
| 5 | 0.022 | 5 | 0.014705882 | 5 | 0.013 |
| 6 | 0.027 | 6 | 0.017647059 | 6 | 0.016 |
| 7 | 0.032 | 7 | 0.020588235 | 7 | 0.018 |
| 8 | 0.036 | 8 | 0.023529412 | 8 | 0.021 |
| 9 | 0.041 | 9 | 0.026470588 | 9 | 0.024 |
| 10 | 0.045 | 10 | 0.029411765 | 10 | 0.026 |
| 11 | 0.050 | 11 | 0.032352941 | 11 | 0.029 |
|  |  | 12 | 0.035294118 | 12 | 0.032 |
|  |  | 13 | 0.038235294 | 13 | 0.034 |
|  |  | 14 | 0.041176471 | 14 | 0.037 |
|  |  | 15 | 0.044117647 | 15 | 0.039 |
|  |  | 16 | 0.047058824 | 16 | 0.042 |
|  |  | 17 | 0.05 | 17 | 0.045 |
|  |  |  |  | 18 | 0.047 |
|  |  |  |  | 19 | 0.050 |

* In each analysis (for each set of outcomes that included biomarkers and individual pathogens), P values were ranked from lowest to highest and compared to the P value cut-off’s shown in order here. An analysis result was only considered statistically significant if it was lower than the corresponding false discovery rate P value cut-off (and if all previous P values from the sequential order for that analysis were also lower than their corresponding false discovery rate P value).

Supplementary Table 4: Proportion of participants with presence of Individual pathogens detected in stool by time point.

|  | **6 months**  Proportion (SD) | **12 months**  Proportion (SD) | **18 months**  Proportion (SD) | **Total sum of proportions** |
| --- | --- | --- | --- | --- |
| EAEC | 0.811 (0.392) | 0.641 (0.480) | 0.487 (0.500) | 1.939 |
| *Giardia* | 0.066 (0.249) | 0.360 (0.480) | 0.613 (0.487) | 1.039 |
| *Campylobacter jejuni* | 0.245 (0.430) | 0.408 (0.492) | 0.273 (0.446) | 0.926 |
| LT ETEC | 0.227 (0.419) | 0.313 (0.464) | 0.285 (0.451) | 0.825 |
| ST ETEC | 0.145 (0.352) | 0.275 (0.447) | 0.178 (0.383) | 0.598 |
| tEPEC | 0.161 (0.368) | 0.204 (0.403) | 0.141 (0.348) | 0.506 |
| *E. bieneusi* | 0.047 (0.213) | 0.236 (0.425) | 0.130 (0.336) | 0.413 |
| *Shigella eiec* | 0.032 (0.177) | 0.215 (0.411) | 0.140 (0.348) | 0.388 |
| *Cryptosporidium* | 0.038 (0.192) | 0.208 (0.406) | 0.124 (0.330) | 0.371 |
| STEC | 0.078 (0.268) | 0.132 (0.338) | 0.155 (0.362) | 0.364 |
| Norovirus | 0.158 (0.365) | 0.108 (0.311) | 0.058 (0.234) | 0.325 |
| Adenovirus | 0.062 (0.241) | 0.044 (0.205) | 0.045 (0.207) | 0.151 |
|  |  |  |  |  |
| Pathogen sum | 2.071 (1.273) | 3.143 (1.493) | 2.636 (1.436) | 7.642 |
|  |  |  |  |  |
| Non-study antimicrobial use | 0.170 (0.376) | 0.152 (0.360) | 0.118 (0.323) |  |

Abbreviations: *EAEC* = enteroaggerative *E. coli*; ETEC = enterotoxigenic *E. coli*; STEC = Shiga toxin-producing *E. coli*; EIEC = enteroinvasive *E. coli*; tEPEC = typical enteropathogenic *E. coli*; *E. bieneusi* = *Enterocytozoon bieneusi*.

Supplementary Figure 1: Proportion of presence of pathogen in stool by month.


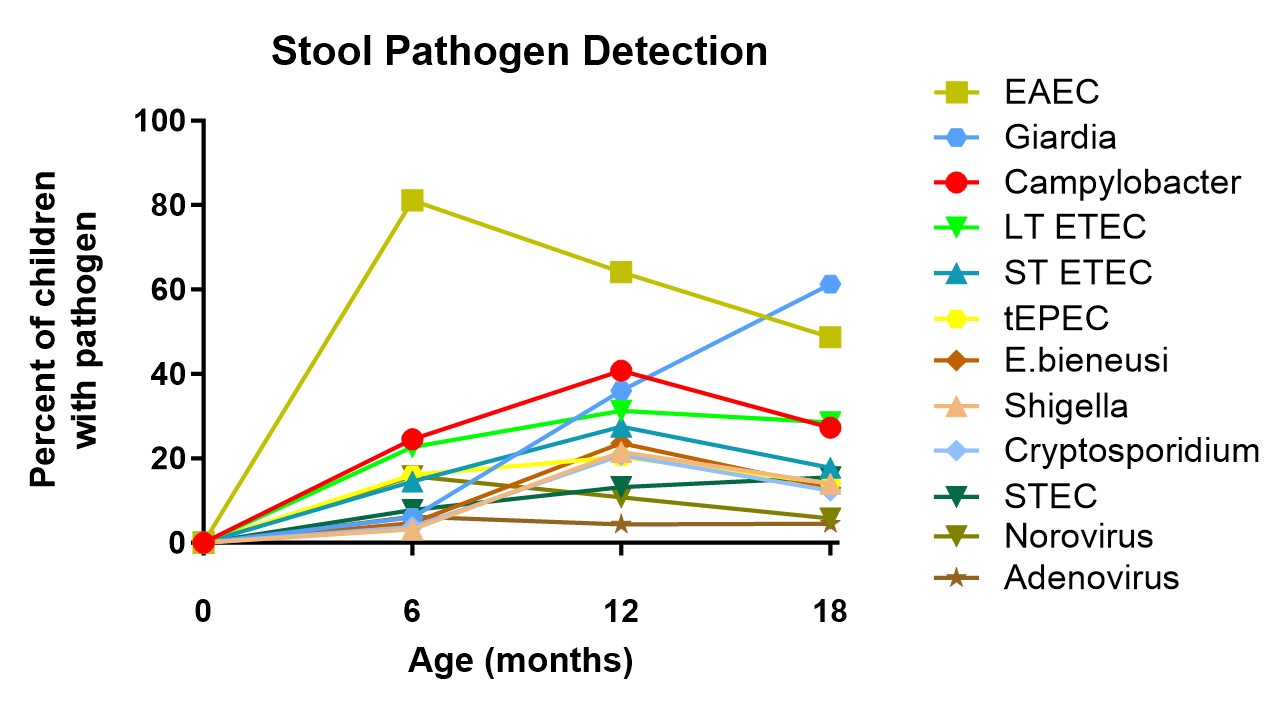


Supplementary Table 5: Predictors the presence of individual organisms in stool samples.*

|  | EAEC | *Giardia* | *Camp. jejuni/coli* | LT ETEC | ST ETEC | tEPEC | *E. bieneusi* | *Shigella*/ EIEC | *Crypto* | STEC | Norovirus | Adenovirus |
| --- | --- | --- | --- | --- | --- | --- | --- | --- | --- | --- | --- | --- |
| Study RCT interventions |  |  |  |  |  |  |  |  |  |  |  |  |
| Antimicrobials | 0.036 (0.048) p=0.459 | 0.059 (0.052) p=0.255 | -0.004 (0.054) p=0.936 | 0.034 (0.042) p=0.416 | 0.071 (0.038) p=0.062 | 0.047 (0.036) p=0.189 | 0.011 (0.035) p=0.744 | -0.016 (0.034) p=0.644 | -0.017 (0.035) p=0.616 | 0.040 (0.033) p=0.233 | 0.048 (0.026) p=0.064 | 0.013 (0.019) p=0.488 |
| Nicotinamide | -0.036 (0.054) p=0.502 | 0.047 (0.057) p=0.406 | -0.041 (0.054) p=0.439 | 0.029 (0.049) p=0.557 | -0.007 (0.044) p=0.874 | -0.033 (0.043) p=0.440 | -0.057 (0.037) p=0.126 | -0.007 (0.036) p=0.855 | 0.048 (0.037) p=0.187 | 0.026 (0.038) p=0.491 | 0.034 (0.036) p=0.340 | -0.019 (0.024) p=0.430 |
|  |  |  |  |  |  |  |  |  |  |  |  |  |
| Non-study antimicrobial past month | 0.424 (0.109) p<0.001 | -0.311 (0.108) p=0.004 | -0.121 (0.109) p=0.268 | 0.109 (0.107) p=0.310 | 0.156 (0.120) p=0.192 | -0.121 (0.135) p=0.370 | -0.144 (0.147) p=0.329 | 0.004 (0.144) p=0.979 | 0.043 (0.147) p=0.770 | -0.266 (0.163) p=0.103 | 0.098 (0.154) p=0.524 | 0.113 (0.214) p=0.598 |
|  |  |  |  |  |  |  |  |  |  |  |  |  |
| Sex | -0.001 (0.054) p=0.989 | 0.066 (0.057) p=0.245 | 0.002 (0.054) p=0.975 | -0.091 (0.049) p=0.065 | -0.012 (0.044) p=0.791 | -0.062 (0.043) p=0.144 | 0.017 (0.037) p=0.649 | -0.029 (0.036) p=0.412 | 0.001 (0.037) p=0.987 | 0.019 (0.038) p=0.614 | -0.067 (0.035) p=0.058 | 0.024 (0.024) p=0.322 |
|  |  |  |  |  |  |  |  |  |  |  |  |  |
| **SES** |  |  |  |  |  |  |  |  |  |  |  |  |
| Mother's education | 0.003 (0.009) p=0.775 | -0.032 (0.009) p=0.001 | -0.022 (0.009) p=0.011 | 0.001 (0.008) p=0.948 | -0.008 (0.007) p=0.257 | -0.002 (0.007) p=0.820 | -0.003 (0.006) p=0.649 | 0.000 (0.006) p=0.949 | -0.013 (0.006) p=0.035 | -0.013 (0.006) p=0.046 | 0.003 (0.006) p=0.564 | 0.000 (0.004) p=0.904 |
| Income | 0.000 (0.001) p=0.743 | -0.003 (0.001) p=0.030 | -0.003 (0.001) p=0.008 | 0.002 (0.001) p=0.061 | -0.003 (0.001) p=0.007 | -0.001 (0.001) p=0.206 | -0.001 (0.001) p=0.228 | -0.001 (0.001) p=0.295 | -0.001 (0.001) p=0.162 | -0.001 (0.001) p=0.291 | 0.000 (0.001) p=0.749 | 0.000 (0.001) p=0.850 |
| WAMI | 0.020 (0.027) p=0.462 | -0.126 (0.028) p<0.0001 | -0.154 (0.027) p<0.0001 | 0.003 (0.025) p=0.895 | -0.026 (0.023) p=0.252 | -0.029 (0.022) p=0.181 | -0.037 (0.019) p=0.050 | -0.035 (0.018) p=0.058 | -0.055 (0.019) p=0.003 | -0.041 (0.019) p=0.035 | -0.017 (0.018) p=0.348 | 0.009 (0.012) p=0.449 |
|  |  |  |  |  |  |  |  |  |  |  |  |  |
| **Housing density** |  |  |  |  |  |  |  |  |  |  |  |  |
| Number siblings | -0.020 (0.012) p=0.098 | 0.022 (0.012) p=0.080 | 0.028 (0.012) p=0.016 | -0.012 (0.011) p=0.269 | 0.007 (0.010) p=0.499 | 0.011 (0.009) p=0.257 | -0.001 (0.008) p=0.902 | 0.012 (0.008) p=0.120 | 0.017 (0.008) p=0.038 | 0.013 (0.008) p=0.136 | -0.002 (0.008) p=0.752 | -0.007 (0.005) p=0.198 |
| Number people in house | -0.011 (0.010) p=0.276 | 0.017 (0.010) p=0.088 | 0.010 (0.010) p=0.294 | 0.007 (0.009) p=0.400 | 0.006 (0.008) p=0.476 | 0.019 (0.008) p=0.012 | 0.006 (0.007) p=0.371 | 0.013 (0.006) p=0.047 | 0.008 (0.007) p=0.227 | 0.009 (0.007) p=0.168 | 0.001 (0.006) p=0.851 | 0.002 (0.004) p=0.681 |
|  |  |  |  |  |  |  |  |  |  |  |  |  |
| **Water/ sanitation** |  |  |  |  |  |  |  |  |  |  |  |  |
| Improved latrine | 0.035 (0.022) p=0.108 | -0.041 (0.023) p=0.081 | -0.052 (0.022) p=0.019 | -0.003 (0.020) p=0.886 | 0.018 (0.018) p=0.334 | 0.009 (0.018) p=0.627 | -0.003 (0.016) p=0.856 | -0.011 (0.015) p=0.455 | -0.021 (0.015) p=0.167 | -0.001 (0.016) p=0.951 | -0.009 (0.015) p=0.547 | 0.011 (0.010) p=0.267 |
| Flush toilet | -0.038 (0.257) p=0.883 | -0.942 (0.274) p=0.001 | -0.530 (0.260) p=0.042 | 0.192 (0.239) p=0.422 | -0.191 (0.216) p=0.377 | 0.185 (0.207) p=0.371 | -0.303 (0.182) p=0.096 | 0.034 (0.175) p=0.845 | -0.183 (0.179) p=0.306 | -0.174 (0.186) p=0.351 | -0.104 (0.179) p=0.561 | 0.044 (0.117) p=0.708 |
| Access to improved water source | 0.004 (0.014) p=0.762 | -0.012 (0.015) p=0.434 | -0.049 (0.014) p=0.001 | -0.016 (0.013) p=0.229 | -0.012 (0.012) p=0.293 | -0.022 (0.011) p=0.053 | -0.014 (0.010) p=0.172 | -0.021 (0.009) p=0.024 | -0.012 (0.010) p=0.205 | -0.014 (0.010) p=0.154 | -0.010 (0.009) p=0.282 | 0.008 (0.006) p=0.217 |
| Water piped into dwelling | 0.063 (0.362) p=0.862 | -1.037 (0.388) p=0.008 | -0.728 (0.366) p=0.047 | -0.212 (0.337) p=0.530 | -0.190 (0.305) p=0.533 | 0.084 (0.292) p=0.774 | 0.000 (0.257) p=0.999 | 0.034 (0.246) p=0.890 | -0.182 (0.252) p=0.471 | 0.028 (0.263) p=0.915 | -0.076 (0.267) p=0.778 | 0.044 (0.166) p=0.792 |
|  |  |  |  |  |  |  |  |  |  |  |  |  |
| **Animals kept at home** |  |  |  |  |  |  |  |  |  |  |  |  |
| Chickens | 0.126 (0.088) p=0.149 | 0.181 (0.091) p=0.047 | 0.129 (0.086) p=0.134 | -0.054 (0.079) p=0.495 | 0.015 (0.072) p=0.835 | -0.044 (0.067) p=0.515 | 0.100 (0.060) p=0.095 | 0.005 (0.057) p=0.936 | 0.023 (0.060) p=0.701 | 0.068 (0.064) p=0.287 | 0.127 (0.058) p=0.028 | 0.041 (0.038) p=0.285 |
| Cows or goats | -0.021 (0.070) p=0.764 | 0.041 (0.074) p=0.579 | 0.009 (0.069) p=0.898 | -0.035 (0.065) p=0.588 | -0.047 (0.057) p=0.405 | 0.083 (0.055) p=0.137 | 0.063 (0.047) p=0.181 | -0.026 (0.046) p=0.582 | -0.080 (0.048) p=0.096 | 0.078 (0.052) p=0.137 | 0.079 (0.047) p=0.090 | -0.001 (0.032) p=0.968 |
| Pigs | -0.132 (0.101) p=0.192 | 0.111 (0.102) p=0.280 | 0.052 (0.097) p=0.594 | 0.007 (0.086) p=0.938 | -0.033 (0.083) p=0.694 | 0.008 (0.078) p=0.918 | 0.071 (0.065) p=0.274 | -0.019 (0.067) p=0.781 | -0.024 (0.065) p=0.712 | 0.013 (0.076) p=0.860 | 0.123 (0.065) p=0.061 | 0.044 (0.045) p=0.330 |
| Agricultural land | -0.193 (0.167) p=0.248 | 0.279 (0.176) p=0.112 | 0.210 (0.166) p=0.207 | 0.052 (0.152) p=0.733 | -0.053 (0.138) p=0.703 | 0.079 (0.132) p=0.551 | 0.082 (0.116) p=0.482 | 0.130 (0.111) p=0.244 | 0.145 (0.114) p=0.204 | 0.012 (0.119) p=0.918 | -0.036 (0.108) p=0.741 | 0.037 (0.075) p=0.617 |

* All tests are linear regression assessing for predictors of the sum of positive tests at months 6, 12 and 18 for individual organism except non-study antimicrobial, which was assessed using mixed model regression for antimicrobial use at months 6, 12, and 18 with the presence of the individual pathogens those months. Analyses assessing for relationships with the antimicrobial intervention were adjusted for 6-month pathogen presence since the intervention did not begin until after the 6-month antimicrobial dose was given. Shaded cells delineate statistical significance by False Discovery Rate (applied to each column individually; see Supplementary Table 3, 17 outcomes).

Abbreviations: *EAEC* = enteroaggerative *E. coli*; ETEC = enterotoxigenic *E. coli*; STEC = Shiga toxin-producing *E. coli*; EIEC = enteroinvasive *E. coli*; tEPEC = typical enteropathogenic *E. coli*; *E. bieneusi* = *Enterocytozoon bieneusi*; *Crypto* = *Cryptosporidium*; SES = socioeconomic status; WAMI = water, assets, maternal education, income

Supplementary Table 6: Cross-sectional associations of number of enteric pathogens at 12 and 18 (separately) with circulating biomarkers, symptoms of illness, and anthropometry at that month. Anthropometry measures are adjusted for baseline measure.

|  | 6 months | P value |  | 12 months | P value |  | 18 months | P value |
| --- | --- | --- | --- | --- | --- | --- | --- | --- |
| Symptoms in previous wk |  |  |  |  |  |  |  |  |
| Illness | 0.96 (0.81, 1.14) | 0.639 |  | 1.06 (0.93, 1.21) | 0.390 |  | 1.00 (0.88, 1.15) | 0.950 |
| Diarrhea | 1.01 (0.85, 1.21) | 0.920 |  | 1.13 (0.97, 1.32) | 0.115 |  | 1.22 (1.03, 1.46) | 0.026 |
| Fever | 1.03 (0.86, 1.23) | 0.765 |  | 1.03 (0.86, 1.24) | 0.722 |  | 1.06 (0.87, 1.30) | 0.566 |
| Sought medical care | 0.98 (0.87, 1.10) | 0.726 |  | 1.07 (0.97, 1.19) | 0.188 |  | 1.05 (0.94, 1.17) | 0.415 |
| Went to hospital | 0.77 (0.44, 1.34) | 0.354 |  | 1.25 (0.85, 1.86) | 0.261 |  | 1.74 (1.01, 2.98) | 0.045 |
|  |  |  |  |  |  |  |  |  |
| Anthropometry |  |  |  |  |  |  |  |  |
| LAZ | -0.017 (0.024) | 0.481 |  | -0.011 (0.020) | 0.573 |  | -0.042 (0.020) | 0.033 |
| WAZ | -0.007 (0.025) | 0.774 |  | -0.051 (0.020) | 0.011 |  | -0.059 (0.020) | **0.003** |
| HCZ | -0.043 (0.023) | 0.059 |  | -0.044 (0.018) | 0.017 |  | -0.057 (0.018) | **0.001** |
|  |  |  |  |  |  |  |  |  |
| Biomarkers |  |  |  |  |  |  |  |  |
| Hemoglobin | NA |  |  | 0.001 (0.023) | 0.974 |  | -0.023 (0.022) | 0.299 |
| Collagen X | NA |  |  | -0.081 (0.024) | **0.001** |  | 0.012 (0.023) | 0.602 |
| IGF-1 | NA |  |  | -0.055 (0.024) | 0.021 |  | -0.102 (0.022) | **<0.0001*** |
| FGF21 | NA |  |  | 0.029 (0.025) | 0.235 |  | 0.086 (0.022) | **<0.0001*** |
| Thyroglobulin | NA |  |  | -0.011 (0.024) | 0.646 |  | 0.007 (0.023) | 0.743 |
| Ferritin | NA |  |  | 0.029 (0.024) | 0.235 |  | -0.021 (0.022) | 0.344 |
| sTFR | NA |  |  | -0.019 (0.025) | 0.432 |  | 0.026 (0.022) | 0.245 |
| RBP4 | NA |  |  | -0.037 (0.025) | 0.145 |  | -0.047 (0.022) | 0.038 |
| CRP | NA |  |  | 0.039 (0.024) | 0.096 |  | 0.059 (0.022) | **0.009*** |
| AGP | NA |  |  | 0.026 (0.024) | 0.279 |  | 0.030 (0.022) | 0.180 |
| CD14 | NA |  |  | -0.007 (0.024) | 0.789 |  | 0.025 (0.023) | 0.260 |

* Bold p-values are statistically significant by False Discovery Rate approach (applied to each column individually; see Supplementary Table 4, 19 outcomes). Abbreviations: LAZ = length-for-age z-score; WAZ = weight-for-age z-score; HCZ = head circumference-for-age z-score; IGF-1 = insulin-like growth factor 1; FGF21 = fibroblast growth factor 21; sTFR = soluble transferrin receptor; RBP4 = retinol binding protein 4; CRP = C-reactive protein; AGP = alpha(1)-acid glycoprotein.

Supplementary Table 7a: Pathogen counts among those with vs. without reported diarrhea by time point.

|  | No diarrhea |  | Diarrhea |  |  |
| --- | --- | --- | --- | --- | --- |
|  | N (%) | Pathogen sum  Mean (SD) | N (%) | Pathogen sum  Mean (SD) | P value |
| 6 months | 977 (92) | 2.072 (1.284) | 81 (8) | 2.086 (1.131) | 0.920 |
| 12 months | 893 (92) | 3.127 (1.500) | 76 (8) | 3.408 (1.416) | 0.115 |
| 18 months | 970 (95) | 2.611 (1.436) | 56 (5) | 3.054 (1.407) | **0.025** |

Supplementary Table 7b: Diarrhea prevalence among those in lower two tertiles vs. upper tertile oof pathogen count, by time point.

|  | Pathogen sum in lower two tertiles | | Pathogen sum in upper tertile | |  |
| --- | --- | --- | --- | --- | --- |
|  | Total  N | With recent diarrhea  N (%) | N (%) | Percent with diarrhea | P value |
| 6 months | 688 | 51 (7.4%) | 370 | 30 (8.0%) | 0.685 |
| 12 months | 586 | 44 (7.5%) | 383 | 32 (8.4%) | 0.632 |
| 18 months | 759 | 35 (4.6%) | 267 | 21 (7.9%) | **0.044** |

Supplementary Table 9a: Mixed-model associations of bacteria and protozoal organisms with symptoms of illness, and anthropometry, and circulating biomarkers.*

|  | EAEC | *Giardia* | *Camp. jejuni/coli* | LT ETEC | ST ETEC | tEPEC | *E. bieneusi* | *Shigella eiec* | *Crypto* | STEC |
| --- | --- | --- | --- | --- | --- | --- | --- | --- | --- | --- |
| Symptoms in previous wk |  |  |  |  |  |  |  |  |  |  |
| Illness | 1.29  (1.04, 1.58), p=0.018 | 0.85  (0.68, 1.07), p=0.164 | 0.92  (0.74, 1.13), p=0.408 | 0.85  (0.69, 1.05), p=0.124 | 1.17  (0.93, 1.47), p=0.179 | 1.12  (0.88, 1.42), p=0.358 | 0.96  (0.72, 1.27), p=0.756 | 1.14  (0.86, 1.51), p=0.354 | 1.32  (1.00, 1.74), p=0.051 | 0.83  (0.61, 1.12), p=0.214 |
| Diarrhea | 1.42  (1.02, 1.96), p=0.036 | 0.80  (0.57, 1.14), p=0.216 | 0.70  (0.50, 0.98), p=0.039 | 0.98  (0.72, 1.33), p=0.887 | 1.40  (1.01, 1.95), p=0.042 | 1.23  (0.87, 1.75), p=0.240 | 0.96  (0.63, 1.47), p=0.863 | 1.80  (1.23, 2.62), p=0.002 | 1.77  (1.20, 2.61), p=0.004 | 0.71  (0.43, 1.16), p=0.166 |
| Fever | 1.61  (1.12, 2.31), p=0.010 | 0.89  (0.61, 1.31), p=0.553 | 0.82  (0.58, 1.16), p=0.265 | 1.12  (0.81, 1.55), p=0.495 | 1.24  (0.86, 1.78), p=0.246 | 0.86  (0.57, 1.30), p=0.463 | 0.88  (0.54, 1.43), p=0.611 | 0.94  (0.57, 1.54), p=0.792 | 1.16  (0.73, 1.85), p=0.523 | 0.91  (0.56, 1.47), p=0.692 |
| Sought medical care | 1.38  (1.12, 1.69), p=0.002 | 0.89  (0.71, 1.10), p=0.281 | 1.02  (0.84, 1.25), p=0.840 | 0.98  (0.80, 1.19), p=0.824 | 1.27  (1.02, 1.58), p=0.031 | 0.87  (0.68, 1.11), p=0.266 | 0.94  (0.71, 1.23), p=0.632 | 1.21  (0.93, 1.59), p=0.162 | 1.27  (0.97, 1.66), p=0.088 | 0.89  (0.67, 1.18), p=0.412 |
| Went to hospital | ** | ** | ** | ** | ** | ** | ** | ** | ** | ** |
| Anthro-pometry |  |  |  |  |  |  |  |  |  |  |
| LAZ | -0.051 (0.029) p=0.086 | -0.076 (0.034) p=0.026 | -0.018 (0.028) p=0.536 | -0.022 (0.030) p=0.466 | -0.009 (0.034) p=0.786 | -0.009 (0.035) p=0.793 | -0.067 (0.039) p=0.088 | -0.026 (0.038) p=0.497 | 0.010 (0.040) p=0.803 | -0.022 (0.039) p=0.577 |
| WAZ | -0.092 (0.026) p=0.001 | -0.089 (0.032) p=0.006 | -0.036 (0.026) p=0.165 | 0.012 (0.025) p=0.638 | -0.047 (0.028) p=0.089 | 0.024 (0.030) p=0.416 | -0.059 (0.032) p=0.071 | -0.124 (0.037) p=0.001 | -0.077 (0.036) p=0.034 | -0.031 (0.034) p=0.353 |
| HCZ | -0.046 (0.026) p=0.081 | -0.039 (0.030) p=0.189 | -0.018 (0.027) p=0.510 | -0.058 (0.027) p=0.036 | -0.084 (0.034) p=0.013 | 0.008 (0.031) p=0.789 | -0.045 (0.032) p=0.159 | -0.116 (0.035) p=0.001 | -0.063 (0.035) p=0.068 | -0.110 (0.040) p=0.006 |
| Biomarkers |  |  |  |  |  |  |  |  |  |  |
| Hemoglobin | 0.025 (0.044) p=0.564 | -0.011 (0.046) p=0.813 | -0.005 (0.046) p=0.906 | -0.026 (0.047) p=0.585 | 0.034 (0.053) p=0.517 | -0.046 (0.058) p=0.428 | 0.011 (0.056) p=0.841 | 0.002 (0.059) p=0.979 | 0.069 (0.061) p=0.260 | -0.060 (0.064) p=0.348 |
| Collagen X | -0.082 (0.048) p=0.091 | 0.013 (0.051) p=0.793 | -0.053 (0.060) p=0.382 | 0.005 (0.047) p=0.921 | -0.070 (0.053) p=0.186 | -0.063 (0.059) p=0.292 | 0.015 (0.053) p=0.781 | -0.203 (0.063) p=0.001 | -0.073 (0.062) p=0.236 | 0.036 (0.057) p=0.530 |
| IGF-1 | -0.046 (0.046) p=0.318 | -0.112 (0.048) p=0.019 | -0.038 (0.046) p=0.415 | -0.065 (0.047) p=0.167 | -0.060 (0.052) p=0.253 | -0.102 (0.058) p=0.079 | -0.141 (0.054) p=0.009 | -0.215 (0.054) p<0.0001 | -0.149 (0.057) p=0.009 | 0.019 (0.063) p=0.756 |
| FGF21 | 0.041 (0.048) p=0.387 | 0.175 (0.049) p<0.001 | -0.015 (0.050) p=0.769 | 0.070 (0.048) p=0.151 | 0.062 (0.058) p=0.282 | 0.040 (0.057) p=0.482 | 0.055 (0.063) p=0.379 | 0.231 (0.062) p<0.001 | 0.082 (0.063) p=0.194 | -0.022 (0.063) p=0.730 |
| Thyroglobulin | 0.031 (0.041) p=0.458 | 0.019 (0.044) p=0.660 | -0.042 (0.042) p=0.315 | -0.020 (0.041) p=0.624 | -0.038 (0.046) p=0.403 | 0.024 (0.053) p=0.655 | 0.006 (0.051) p=0.908 | -0.082 (0.054) p=0.133 | 0.019 (0.049) p=0.695 | -0.022 (0.049) p=0.654 |
| Ferritin | -0.003 (0.048) p=0.950 | 0.063 (0.047) p=0.182 | -0.037 (0.048) p=0.445 | -0.041 (0.047) p=0.391 | -0.016 (0.055) p=0.764 | 0.067 (0.065) p=0.305 | -0.071 (0.059) p=0.230 | 0.062 (0.061) p=0.309 | 0.045 (0.058) p=0.433 | 0.033 (0.063) p=0.601 |
| sTFR | -0.015 (0.046) p=0.746 | 0.006 (0.048) p=0.900 | 0.017 (0.046) p=0.717 | -0.016 (0.048) p=0.745 | -0.071 (0.051) p=0.163 | -0.014 (0.059) p=0.816 | -0.017 (0.056) p=0.761 | -0.079 (0.058) p=0.173 | -0.069 (0.055) p=0.204 | 0.068 (0.056) p=0.222 |
| RBP4 | 0.043 (0.051) p=0.403 | -0.008 (0.049) p=0.879 | 0.033 (0.051) p=0.511 | -0.090 (0.050) p=0.070 | -0.114 (0.061) p=0.063 | -0.044 (0.058) p=0.449 | -0.263 (0.066) p<0.0001 | -0.049 (0.066) p=0.461 | 0.014 (0.066) p=0.826 | 0.014 (0.060) p=0.821 |
| CRP | 0.092 (0.049) p=0.060 | 0.109 (0.048) p=0.023 | 0.061 (0.050) p=0.218 | 0.050 (0.051) p=0.330 | 0.053 (0.057) p=0.359 | 0.055 (0.064) p=0.392 | -0.020 (0.061) p=0.744 | 0.157 (0.062) p=0.011 | 0.020 (0.062) p=0.749 | 0.020 (0.062) p=0.746 |
| AGP | -0.012 (0.049) p=0.803 | 0.120 (0.048) p=0.013 | -0.030 (0.050) p=0.547 | 0.007 (0.050) p=0.881 | 0.055 (0.055) p=0.316 | 0.044 (0.062) p=0.479 | -0.046 (0.060) p=0.440 | 0.144 (0.061) p=0.019 | 0.027 (0.062) p=0.665 | 0.064 (0.062) p=0.309 |
| CD14 | 0.001 (0.051) p=0.977 | 0.156 (0.050) p=0.002 | -0.048 (0.053) p=0.361 | 0.002 (0.053) p=0.971 | -0.056 (0.052) p=0.279 | -0.037 (0.065) p=0.573 | -0.052 (0.057) p=0.364 | 0.020 (0.061) p=0.741 | 0.059 (0.071) p=0.409 | -0.002 (0.060) p=0.980 |

* Model included sex, SES (WAMI), age and birthseason. Shaded cells are statistically significant by False Discovery Rate approach (applied to each column individually; see Supplementary Table 3, 19 outcomes).

** Mixed model did not converge.

Abbreviations: *EAEC* = enteroaggerative *E. coli*; ETEC = enterotoxigenic *E. coli*; STEC = Shiga toxin-producing *E. coli*; EIEC = enteroinvasive *E. coli*; tEPEC = typical enteropathogenic *E. coli*; *E. bieneusi* = *Enterocytozoon bieneusi*; *Crypto* = *Cryptosporidium*; IGF-1 = insulin-like growth factor 1; FGF21 = fibroblast growth factor 21; sTFR = soluble transferrin receptor; RBP4 = retinol binding protein 4; CRP = C-reactive protein; AGP = alpha(1)-acid glycoprotein.

Supplementary Table 9b: Mixed-model associations of individual viruses with symptoms of illness, and anthropometry, and circulating biomarkers.*

|  | Norovirus | Adenovirus |
| --- | --- | --- |
| Symptoms in previous wk |  |  |
| Illness | 1.14  (0.86, 1.52), p=0.359 | 0.91  (0.59, 1.40), p=0.671 |
| Diarrhea | 1.74  (1.19, 2.56), p=0.005 | 1.40  (0.78, 2.50), p=0.255 |
| Fever | 1.42  (0.93, 2.18), p=0.109 | 0.71  (0.32, 1.55), p=0.386 |
| Sought medical care | 1.06  (0.79, 1.41), p=0.712 | 1.20  (0.81, 1.78), p=0.372 |
| Went to hospital | ** | ** |
| Anthro-pometry |  |  |
| LAZ | -0.047 (0.045) p=0.304 | 0.002 (0.062) p=0.977 |
| WAZ | 0.026 (0.041) p=0.532 | -0.002 (0.058) p=0.969 |
| HCZ | 0.039 (0.041) p=0.343 | 0.038 (0.066) p=0.569 |
|  |  |  |
| Hemoglobin | 0.076 (0.073) p=0.297 | 0.035 (0.103) p=0.736 |
| Collagen X | -0.106 (0.086) p=0.219 | -0.071 (0.103) p=0.494 |
| IGF-1 | -0.323 (0.068) p<0.0001 | 0.231 (0.113) p=0.041 |
| FGF21 | 0.048 (0.096) p=0.614 | -0.060 (0.113) p=0.597 |
| Thyroglobulin | -0.030 (0.066) p=0.654 | -0.016 (0.091) p=0.858 |
| Ferritin | -0.024 (0.093) p=0.799 | 0.028 (0.097) p=0.772 |
| sTFR | 0.016 (0.075) p=0.833 | -0.013 (0.086) p=0.883 |
| RBP4 | -0.171 (0.108) p=0.116 | -0.024 (0.109) p=0.822 |
| CRP | 0.150 (0.094) p=0.109 | -0.083 (0.110) p=0.450 |
| AGP | -0.083 (0.076) p=0.277 | 0.018 (0.125) p=0.884 |
| CD14 | 0.128 (0.093) p=0.173 | -0.022 (0.135) p=0.874 |

* Model included sex, SES (WAMI), age and birthseason. Shaded cells are statistically significant by False Discovery Rate approach (applied to each column individually; see Supplementary Table 3, 19 outcomes).

** Mixed model did not converge.

Abbreviations: LAZ = length-for-age z-score; WAZ = weight-for-age z-score; HCZ = head circumference-for-age z-score; IGF-1 = insulin-like growth factor 1; FGF21 = fibroblast growth factor 21; sTFR = soluble transferrin receptor; RBP4 = retinol binding protein 4; CRP = C-reactive protein; AGP = alpha(1)-acid glycoprotein.

Supplementary Table 8: Causal mediation analysis assessing biomarkers as potential mediators between pathogen burden and anthropometry outcomes. This analysis was performed for biomarkers that were significantly associated with pathogen burden.

|  | LAZ |  |  |  | WAZ |  |  |  | HCZ |  |  |
| --- | --- | --- | --- | --- | --- | --- | --- | --- | --- | --- | --- |
| Mediator | Estimate | Proportion attributed to mediator | p-value |  | Estimate | Proportion attributed to mediator | p-value |  | Estimate | Proportion attributed to mediator | p-value |
| Collagen X | -0.00143 | 0.0618 | 0.204 |  | -0.00277 | 0.0680 | **0.028** |  | -0.00125 | 0.0225 | 0.078 |
| IGF-1 | -0.00395 | 0.18953 | 0.146 |  | -0.00617 | 0.1515 | **<2e-16** |  | -0.00322 | 0.0624 | 0.066 |
| FGF21 | -0.00273 | 0.12723 | 0.25 |  | -0.00792 | 0.2014 | **<2e-16** |  | -0.00176 | 0.0354 | 0.25 |
| CRP | -0.00025 | 0.006717 | 0.648 |  | -0.00035 | 0.0059 | 0.47 |  | 0.000248 | -0.0027 | 0.6 |

Abbreviations: LAZ = length-for-age z-score; WAZ = weight-for-age z-score; HCZ = head circumference-for-age z-score; IGF-1 = insulin-like growth factor 1; FGF21 = fibroblast growth factor 21; CRP = C-reactive protein

Supplementary Table 9c: Mixed-model associations of non-study antimicrobials with symptoms of illness, and anthropometry, and circulating biomarkers.

|  | All non-study antimicrobials | Macrolides | Cephalosporins | Sulfonamides | Penicillins | Metronidazole | Fluoroquinolones |
| --- | --- | --- | --- | --- | --- | --- | --- |
| Symptoms in previous wk* |  |  |  |  |  |  |  |
| Illness | 25.35  (19.73, 32.56) p<0.0001 | 23.88  (10.39, 54.88) p<0.0001 | † | 5.58  (2.93, 10.62) p<0.0001 | 18.69  (13.82, 25.26) p<0.0001 | 14.18  (9.25, 21.73) p<0.0001 | 8.80  (0.73, 106.06) p=0.087 |
| Diarrhea | 12.29 (9.09, 16.64)  p<0.0001 | 9.24  (4.81, 17.77) p<0.0001 | 4.58  (0.89, 23.57) p=0.069 | 7.96  (4.07, 15.55) p<0.0001 | 3.37  (2.36, 4.80) p<0.0001 | 28.95  (18.97, 44.19) p<0.0001 | 8.05  (0.62, 104.01), p=0.110 |
| Fever | 12.68 (9.18, 17.52)  p<0.0001 | 12.98  (6.80, 24.78) p<0.0001 | 11.03  (2.73, 44.58) p=0.001 | 2.84  (1.19, 6.80) p=0.019 | 11.43  (8.20, 15.93) p<0.0001 | 3.33  (1.97, 5.63) p<0.0001 | 8.24  (0.70, 96.76), p=0.093 |
| Sought medical care | † | † | † | † | † | † | † |
| Went to hospital | † | † | † | † | † | † | † |
|  |  |  |  |  |  |  |  |
| Anthropometry at 18 mo** |  |  |  |  |  |  |  |
| LAZ | 0.017 (0.014) p=0.227 | -0.036 (0.061) p=0.554 | -0.198 (0.161) p=0.220 | -0.137 (0.052) p=0.009 | 0.066 (0.019) p=0.001 | -0.003 (0.038) p=0.944 | -0.028 (0.154) p=0.854 |
| WAZ | 0.009 (0.014) p=0.532 | -0.079 (0.057) p=0.163 | -0.339 (0.158) p=0.032 | -0.038 (0.057) p=0.503 | 0.045 (0.020) p=0.025 | -0.022 (0.035) p=0.540 | 0.148 (0.206) p=0.472 |
| HCZ | 0.007 (0.012) p=0.571 | -0.055 (0.050) p=0.271 | -0.153 (0.161) p=0.345 | -0.028 (0.053) p=0.597 | 0.023 (0.018) p=0.182 | 0.004 (0.031) p=0.892 | 0.062 (0.173) p=0.719 |
| Biomarkers*** |  |  |  |  |  |  |  |
| Hemoglobin | 0.004 (0.017) p=0.835 | 0.079 (0.076) p=0.294 | -0.089 (0.199) p=0.654 | -0.167 (0.073) p=0.023 | 0.044 (0.023) p=0.059 | -0.019 (0.112) p=0.865 | 0.452 (0.284) p=0.112 |
| Collagen X | 0.009 (0.019) p=0.624 | 0.121 (0.064) p=0.061 | 0.021 (0.137) p=0.879 | -0.068 (0.077) p=0.380 | 0.021 (0.027) p=0.449 | -0.293 (0.104) p=0.005 | -0.123 (0.223) p=0.582 |
| IGF-1 | 0.025 (0.016) p=0.117 | -0.026 (0.073) p=0.725 | 0.071 (0.187) p=0.705 | -0.044 (0.064) p=0.489 | 0.041 (0.023) p=0.074 | -0.117 (0.099) p=0.235 | 0.554 (0.257) p=0.031 |
| FGF21 | -0.021 (0.020) p=0.286 | 0.102 (0.083) p=0.216 | -0.337 (0.212) p=0.111 | -0.124 (0.077) p=0.106 | -0.028 (0.027) p=0.305 | 0.153 (0.107) p=0.153 | -0.512 (0.257) p=0.046 |
| Thyroglobulin | 0.003 (0.018) p=0.876 | -0.067 (0.071) p=0.344 | -0.284 (0.136) p=0.038 | 0.034 (0.080) p=0.673 | 0.032 (0.027) p=0.232 | -0.032 (0.080) p=0.692 | -0.290 (0.241) p=0.228 |
| Ferritin | 0.009 (0.019) p=0.627 | 0.026 (0.077) p=0.739 | 0.159 (0.244) p=0.514 | -0.043 (0.071) p=0.543 | 0.028 (0.027) p=0.297 | 0.007 (0.121) p=0.956 | 0.167 (0.264) p=0.528 |
| sTFR | -0.031 (0.018) p=0.082 | -0.082 (0.077) p=0.287 | 0.122 (0.255) p=0.633 | 0.015 (0.067) p=0.820 | -0.067 (0.026) p=0.009 | -0.142 (0.093) p=0.128 | -0.262 (0.186) p=0.158 |
| RBP4 | 0.014 (0.018) p=0.416 | -0.061 (0.070) p=0.380 | 0.495 (0.143) p=0.001 | -0.073 (0.062) p=0.236 | 0.044 (0.026) p=0.094 | -0.227 (0.107) p=0.034 | 0.156 (0.236) p=0.509 |
| CRP | 0.002 (0.018) p=0.918 | 0.017 (0.068) p=0.802 | -0.179 (0.135) p=0.184 | 0.124 (0.064) p=0.052 | 0.000 (0.027) p=0.997 | -0.180 (0.112) p=0.109 | 0.047 (0.354) p=0.893 |
| AGP | -0.003 (0.018) p=0.855 | 0.130 (0.080) p=0.103 | -0.314 (0.116) p=0.007 | 0.036 (0.069) p=0.602 | -0.013 (0.026) p=0.626 | 0.044 (0.131) p=0.737 | 0.211 (0.281) p=0.454 |
| CD14 | -0.043 (0.017) p=0.010 | -0.024 (0.079) p=0.765 | 0.120 (0.115) p=0.295 | -0.077 (0.065) p=0.239 | -0.060 (0.024) p=0.012 | -0.239 (0.108) p=0.026 | 0.273 (0.169) p=0.108 |

Shaded cells are statistically significant by False Discovery Rate approach (applied to each column individually; see Supplementary Table 3, 19 outcomes).

* Mixed model logistic regression (proc glimmix in SAS) adjusted for sex, SES (WAMI), birth month and age. Odds ratio and 95% confidence interval are shown.

** Linear regression of number of antimicrobial doses from birth through 18 months on anthropometry measure at 18 months, adjusted for baseline anthropometry measure, sex, SES (WAMI), and birth month. Estimate and standard error are shown.

*** Linear regression of number of antimicrobial doses from birth through 18 months on mean value of biomarker from 12 and 18 months, adjusted for sex, SES (WAMI), and birth month. Estimate and standard error are shown.

† Mixed regression model did not converge.

Supplementary Figure 2: Concept figure displaying associations between predictors of pathogen burden, pathogen count and outcomes and potential etiologies.


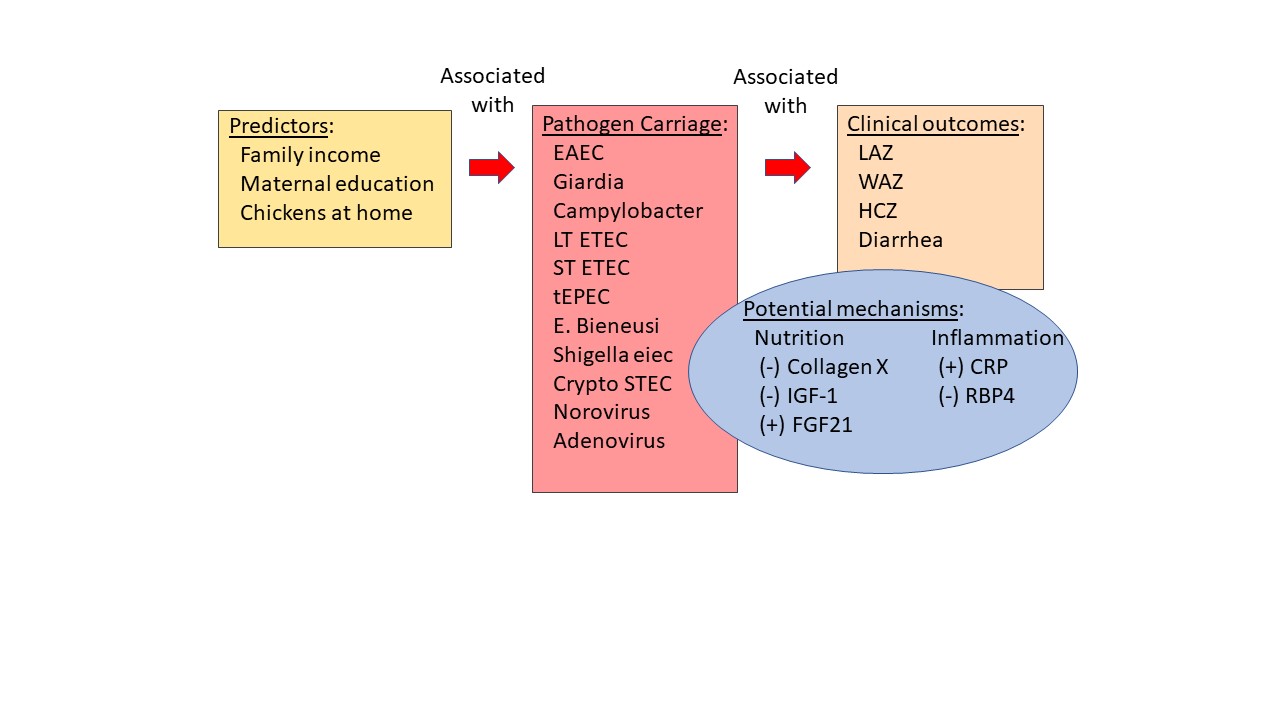

Supplement: online supplemental file 1 [file bmjgh-10-11-s001.docx]
